# Shipbuilding 4.0 Index Approaching Supply Chain

**DOI:** 10.3390/ma12244129

**Published:** 2019-12-10

**Authors:** Magdalena Ramirez-Peña, Francisco J. Abad Fraga, Alejandro J. Sánchez Sotano, Moises Batista

**Affiliations:** 1 Department of Mechanical Engineering and Industrial Design, School of Engineering, University of Cádiz, Av. Universidad de Cádiz, 10, E-11519 Puerto Real (Cádiz), Spain; alejandrojavier.sanchez@uca.es; 2 Navantia S.A., SME Astillero Bahía de Cádiz, Polígono Astilleros s/n, E-11519 Puerto Real (Cádiz), Spain; fabad@navantia.es

**Keywords:** shipbuilding, LARG paradigm, supply chain

## Abstract

The shipbuilding industry shows a special interest in adapting to the changes proposed by the industry 4.0. This article bets on the development of an index that indicates the current situation considering that supply chain is a key factor in any type of change, and at the same time it serves as a control tool in the implementation of improvements. The proposed indices provide a first definition of the paradigm or paradigms that best fit the supply chain in order to improve its sustainability and a second definition, regarding the key enabling technologies for Industry 4.0. The values obtained put shipbuilding on the road to industry 4.0 while suggesting categorized planning of technologies.

## 1. Introduction

Every industrial revolution has brought important improvements in terms of manufacturing. Since 2015, the industry is working on the so-called fourth industrial revolution or Industry 4.0. This fourth industrial revolution introduces new advanced production models with new technologies that allow the digitalization of processes, services, and even business models [1]. Among other innovations, Industry 4.0 (I4.0) gives rise to the inclusion of the social aspect in the definition of the performance model of manufacturing processes, thus completing the economic, energy, environmental, and functional aspects considered until now [2].

It is also very common nowadays to use the distributed manufacturing systems which consist of manufacturing components in different physical locations and then going through supply chain management, bringing them together for the final assembly of a complex product [3]. Within the shipbuilding industry, there are two distinct fields of work, one dedicated to the repair, maintenance, or improvement of ships already built and the second dedicated to new ships. Focusing on the new construction and referring to it as shipbuilding could be consider as a case of distributed manufacturing, where the different blocks that constitute the ship built in different workshops belonging to the same manufacturing center are assembled afterwards in the dock. Therefore, shipbuilding is a complex manufacturing process that must adapt to I4.0 in order to progress. In this case, shipbuilding is a complex industry, with a complex structure composed of a large number of suppliers belonging to different locations, sizes, and typologies [4]. In addition, any small change made by each part of this structure not only affects the rest of the members but can also have enormous consequences. In this type of complex manufacturing [5], supply chain (SC) is a key factor to improve the efficiency of the shipyard in adapting to I4.0 [6]. Thus, the digitization—objective of the I4.0—of the supply chain will provide it with the agility and efficiency that shipbuilding needs to be more profitable [7,8].

Supply chain is the set of the flows of materials and information that take place within a company from the suppliers of raw materials to the consumer of the final product [9]. It is the concept that connects companies to their suppliers [10], as well as having among its activities the control of logistic activities [11] and the responsibility of analyzing purchases [12]. Supply chain represents one of the areas with the greatest investment in successful companies as it has become a strategic tool with a multidisciplinary and transversal character that affects all strategic levels of the company. It affects the sector and the market where the company is going to compete, defined by the corporate strategy, how it is going to compete, defined by the competitive strategy and of course each of the affected areas within the company, defined by the functional strategy [13].

Within the objectives of the supply chain are a rapid response to demand, flexible manufacturing, cost reduction, and inventory reduction. In addition, through the achievement of these objectives, SC aims to achieve improved competitiveness and sustainability of the company [14]. In the framework of Industry 4.0, the main objective of the supply chain must be total visibility of all product movements for each member of the chain as well as a total integration [15]. The most important paradigms on the supply chain found in the literature, under the perspective of sustainability shows the paradigm LARG. It is the paradigm defined under the acronym LARG: Lean, Agile, Resilient, and Green [14].

Lean Paradigm: The principles on which the Lean philosophy is based and its practices, make them ideal for the supply chain consisting of a network of business units or even independent companies, becoming challenging because of the complexity of management [16]. Among its contributions are:

- A collaborative relationship between its members of mutual trust and long-lasting commitment.
- Few and closer suppliers with low vertical integration are preferred.
- Multifaceted criteria approach is recommended on the capacity and benefit of suppliers and on the previous relationship.
- Software development for suppliers.
- The involvement of suppliers from the early stages of new product design and development processes.
- Frequent feedback allows risks, benefits, and solutions to be shared [17].

Agile Paradigm: The agile supply chain must know what is happening in the market in order to be able to respond as quickly and close as possible to reality [18]. It is through the integration of partners where the acquisitions of new skills allow them to respond quickly to the constant changes in the market [19]. The key elements are their dynamic structure and the visibility of information configured from beginning to end of an event-based management, such as relationships. For some authors, the supply chain should be adjusted when there is a question of a production of a considered volume with little variety, in a predictable, controllable business environment, whereas, if it is a question of unpredictable changes in the market, a small volume and a great variety are required, in this case an agile paradigm is required. Other authors such as Naylor et al. [20] introduce the term “Leagile” for the supply chain whenever demand is variable and there is a wide variety of products.

Resilient Paradigm: Resilience is the ability to overcome the disturbances suffered and recover the state in which it was before the disturbance. Based on this definition, the supply chain must have this characteristic and understand resilience as the capacity that the organization must have to continuously adjust the supply chain of events that may alter the balance of its activities [21]. One of the objectives of the resilient supply chain is to avoid a change to an undesirable state [14]. One way to achieve this is to design strategies to restore the previous state of the system [22]. Among the must have characteristics resilient supply chain, most authors agree on the total visibility of it; characteristic shared with the Agile paradigm and with Industry 4.0 [23,24]. However, several authors consider this paradigm very costly and complicated to implement. Therefore, they consider Lean Production and/or Six Sigma option as an alternative that provides flexibility and a corporate culture that could also provide resilient to supply chain [25].

Green Paradigm: This paradigm offers different approaches, from the perspective of supplier management in terms of risks and returns, in terms of supply chain management for sustainable products or both at the same time [26]. This ecological supply chain term is the consideration of environmental extension within supply chain management from the stages of product design, to the manufacturing process itself, until the delivery to the final consumer and even to the end-of-life management of the manufactured product [27]. This paradigm even lead the determination that through the greening of the different stages of the supply chain an integrated green supply chain can be achieved, which would lead to an increase in competitiveness and economic performance [28,29]. Sustainable supply chain also consider coordination of economic, environmental, and social considerations [30,31]. 

Looking for quantifiers on the supply chain in the literature, different parameters analysis are detected. Of those who seek the measure of their performance, some do so quantitatively, others qualitatively and there are those who analyze from both perspectives, identifying parameters such as visibility as Lia et al. did [6]. There are approaches to improving supply chain performance at different stages of the product life cycle by applying different linguistic scales to assess uncertain supply behavior or as in the case of Chang et al. [32] studying the selection of suppliers using fuzzy logic. This approach allows companies the assessment with no limitation on categories of scale and data [33]. However, there are other approaches such as improving the decision-making process. Wang et al. researched to provide decision-makers with rapid access to the practical performance of suppliers supply [34].

Alternatively, and as decision support process for incomplete hesitant fuzzy preference relations, the qualification of the supply chain is evaluated [35]. Other studies show focus on relieving the complexity of the aggregation and evaluation process by showing the connection between product strategies and supply chain performance [36]. In addition, there are benchmarking tools that develop indices to measure the agility of the supply chain such as Lin et al. [37]. Also to evaluate parameters of the supply chain itself as green or resilient [38] or even several at once as is the case of the LARG index, which evaluates the supply chain from the perspective of Lean, Agile, Resilient, and Green researched by Azevedo et al. [39]. 

In this case, the aim is to evaluate a shipyard in the process of adapting to industry 4.0 through its supply chain. There are already studies in which a conceptual model is being developed that separately confronts the different paradigms that make up the LARG paradigm and confronts them with the enabling technologies of Industry 4.0 in the field of shipbuilding [40]. In the case at hand and based on the previous studies of Azevedo [38,39], the Delphi method is used as a strategic information-gathering tool considered appropriate for supply chain [39]. The Delphi method consists of the technique that allows information gathered through consultation with experts [39]. It is an iterative process based on the anonymity of the answers, which allows the analysis of the answers. It is composed of several phases: 1. Definition of the research problem, 2. Determination of the participants to take part, 3. Elaboration of the questionnaire establishing the number of necessary rounds, and 4. Results [41]. A small number of participants (6–30) makes this technique best suited to scientific evidence and social values [42], coinciding with the new aspect that introduces the I4.0 in the economic performance of companies [43]. 

There is no previous experience of quantifying the contribution of the LARG paradigm in the shipbuilding industry, only the experience of Azevedo in the automotive industry [39], or even of the 4.0 industry in general. However, knowing that SC determine KETs is possible to look for the most important practices associated with each technology for each paradigm of the supply chain. Analyzing these practices, the relationship between the technologies and the supply chain is analyzed at the same time. Then the method developed allows evaluating how LARG is the shipbuilding industry related to its practices. In the second phase, in order to know how 4.0 SC is through the evaluation of the implementation of KETs according to each of them to the supply chain paradigms. In this case, in addition to carrying out both quantifications, a special index is created to know how advanced is the adaptation to the 4.0 industry of the shipbuilding supply chain, this being the main objective of this paper. Based on all the above, the importance of the supply chain to a company remains latent, especially for companies as complex as those dedicated to shipbuilding, specifically to the shipyard. The purpose of the article is to define an index that shows the situation in which a shipbuilding company has in relation to its adaptation to Industry 4.0, addressing its supply chain. The proposal is, on first place to define the paradigms that best suit the achievement of sustainability in the company. Second, analyze how each of the enabling technologies of Industry 4.0 affects the supply chain through the evaluation of the results obtained with the Delphi method. This evaluation of results will allow the shipyard to establish under which paradigms of the supply chain to work as well as to know which technologies will allow it to fully adapt to industry 4.0. 

## 2. Experimental Methodology

**Figure 1:**
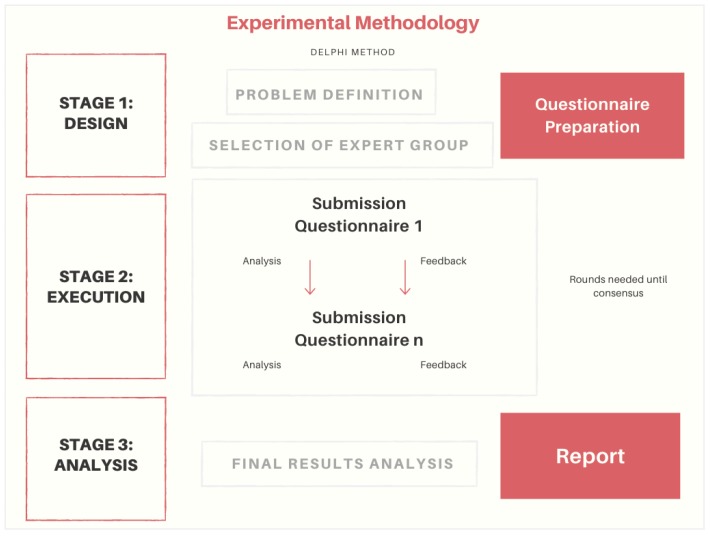
Scheme of the experimental methodology followed.

As already mentioned, the experimental methodology follows the Delphi method. Previously, the Delphi method has already been used in issues related to supply chain. A collaboration index between retailers and manufacturers studied by Anbanandam et al. [44], Supply Chain Fragility Index by Stonebraker et al. [45], performance SC index proposed by Nunlee et al. [46], or risk assessment index studied by Rao and Schoenherr [47]. In this communication, the proposal is to develop a shipbuilding 4.0 index. Figure 1 shows a general diagram developed below:

### 2.1. Stage 1: Design

The first stage of the experimental methodology begins with the definition of the problem, the selection of experts according to the problem addressed and the definition of the appropriate questionnaire that allows us to reach the solution of the problem posed.

Regarding the definition of the problem, the aim is to assess the level of adaptation of a shipbuilding company, specifically dedicated to the block assembly, to the 4.0 industry by addressing its supply chain. To this end, the first step is to define one or more of the supply chain paradigms that are most appropriate for this type of company in such a complicated sector. Specifically, the aim is to study the “LARG” paradigm formed by the combination of the Lean, Agile, Resilient, and Green paradigms.

At the same time, this stage has to establish which of the enabling technologies for Industry 4.0 facilitates and are best suited to contribute to the sustainability supply chain and subsequent implementation in the case study. There are studies that establish that there are twelve technologies that are suitable for shipbuilding [40]. These technologies are Additive Manufacturing, Big Data, Cloud Computing, Augmented Reality, Autonomous Robots, Automatic Guided Vehicles, Blockchain, Cybersecurity, Horizontal and Vertical Integration System, Artificial Intelligence, Internet of things and Simulation.

A very important weight in the design lies on the selection of experts to participate in the surveys. It is ideal to select people who are interested in the subject matter and whose expertise includes the topic in question, as well as their impartiality. At this point, forty people take part in the survey. Twenty of them are personnel from the shipbuilding sector itself, as well as twenty from academics directly related to the sector. In this sense, one can have essential perspectives on the object of research. Finally, half of all guests agreed to participate.

It is now possible to define the questions that will constitute the questionnaire. It has seventeen sections. A first section in which an assessment is requested from nothing important to extremely important, where nothing important is weighted with 1 point and extremely important with 5 points, on the importance of each of the Supply Chain paradigms, Lean, Agile, Resilient and Green. Table 1 shows the questions in section 1 as well as the average rating values and weights of each of the paradigms posed.

Sections two to five evaluate the implementation or non-implementation of four practices of each of the paradigms. Table 2 shows questions sections 2 to 5, indicated by P for Practice, sub-indices L, A, R, G, followed by numbering from 1 to 4, mean rating values and weightings of these practices. Finally, sections six to seventeen include the weighting of the importance of each of the twelve technologies, according to each of the supply chain paradigms. Table 3 shows the questions in sections 6 to 17 that highlight the importance of each of the industry 4.0 enabling technologies for the shipbuilding supply chain.

### 2.2. Stage 2: Execution

Once the questionnaire design is complete, it is ready to launch the first round of surveys to all professionals who have agreed to participate. This is sent virtually, via e-mail, where the survey is in a form linked in that e-mail.

After the first round, data is collected and analyzed. To know the level of agreement reached by the experts, the Kendall concordance coefficient is used. This coefficient indicates that the level of agreement reached in this first round is low. It is for this reason that it is necessary to carry out a new round.

In the same way, a second round is sent, in which the participants are informed of the mean rating results of the first round. In this second round, the Kendall correlation coefficient has increased in value indicating that there is greater consistency between the professionals and academics. At this point, it is decided to conclude the surveys and proceed to analyze them.

### 2.3. Stage 3: Analysis

Data analysis has three phases. In the first one, the importance of each supply chain paradigm is analyzed separately, i.e., the importance of the Lean, Agile, Resilient, and Green paradigms for the shipbuilding supply chain. The second phase analyses how each of the previous paradigms has been implemented. After knowing this value, we obtain a general index that will allow us to know how LARG shipbuilding supply chain is.

It is in the third phase and through the twelve key enabling technologies (KETs), where we get to know the importance of each of the KETs for the supply chain of shipbuilding. In the three phases, using the same mathematical procedure, the mean rating, weightings, and consequent indices mentioned are calculated.

## 3. Results

### 3.1. How Important is Each LARG Paradigm to Shipbuilding Supply Chain

**Figure 2:**
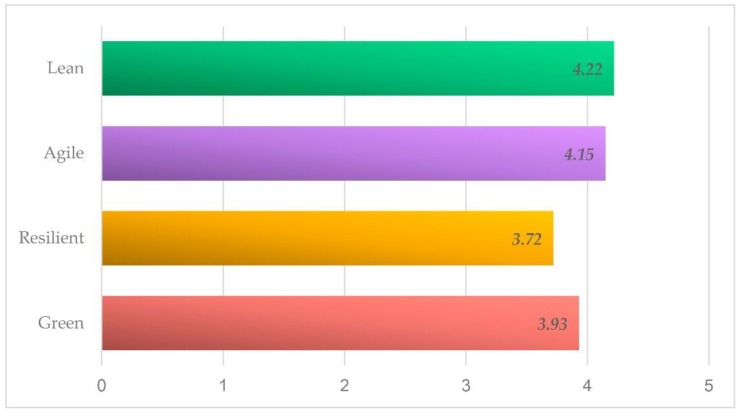
Importance of LARG to Shipbuilding SC.

The first result found presents the importance of the Lean, Agile, Resilient, and Green paradigms. As a result of the mean rating, the weight for each paradigm is calculated according to the equation [58]:

(1) $$w_{x}=\frac{\mathrm{Mx}}{\sum_{g=1}^{n}\mathrm{Mg}}$$

where $w_{x}$ represents the weighting of the paradigm x, Mx represents the mean rate of that particular paradigm and $\;\overset{n}{\underset{g=1}{\sum }}\mathrm{Mg}$ represents the sum of the means for each paradigm. Figure 2 shows the relative results obtained by studying the importance of LARG for Shipbuilding Supply Chain represented by its mean rating. Table 1 shows the questions in section 1, mean rating values and weightings of each paradigms asked.

As can be seen, the Lean paradigm is the most valued of all; followed closely by the Agile paradigm, even more than the Green paradigm despite the importance it must have for this sector.

### 3.2. How Implanted is Each LARG Paradigm to Shipbuilding Supply Chain

**Figure 3:**
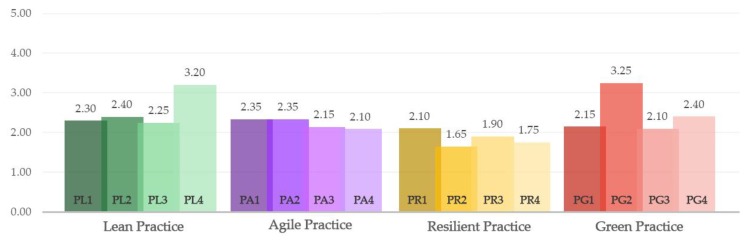
Level of implantation of LARG to shipbuilding SC.

In the same way, with the values contributed by the experts with respect to the level of implantation, we are in disposition to calculate how much is each one of the studied paradigms is implemented. Figure 3 represents the level of implementation of LARG for shipbuilding supply chain represented by its mean rating. Table 2 shows the questions sections 2 to 5, indicated by P for Practice, sub-indices L, A, R, G, followed by numbering from 1 to 4, mean rating values, and weightings of these practices.

It is now defined the expert behavior as:

(2) $${\mathrm{EB}}_{i}=\overset{n}{\underset{i=1}{\sum }}\left(D_{\mathrm{ixj}}\cdot w_{\mathrm{Pxj}}\right)$$

where D_ixj_ is the answer of the expert i to practice j of the paradigm x. At the same time, the behavior of each paradigm is:

(3) $${\mathrm{PB}}_{x}=\frac{\sum_{i=1}^{n}\left({\mathrm{EB}}_{i}\right)}{n}$$

**Figure 4:**
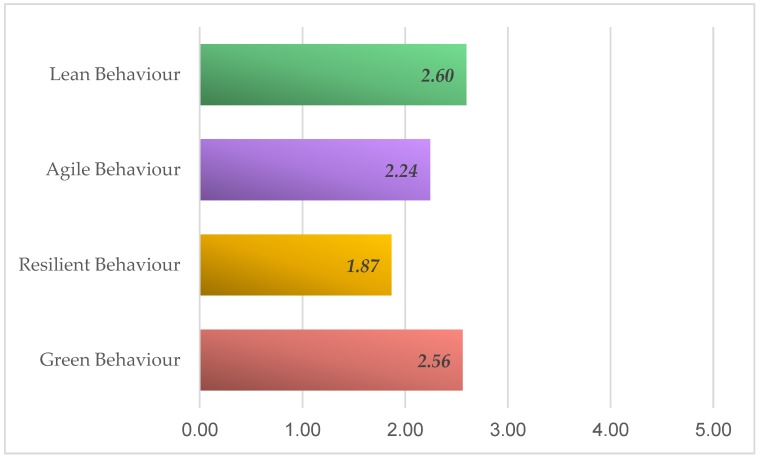
Paradigms behavior.

Figure 4 shows the results obtained.

With these data, it is possible to calculate the implementation of each paradigm for the supply chain (SCx):

(4) $${\mathrm{SC}}_{x}={\mathrm{PB}}_{x}\cdot w_{x}$$

Being LARG_SC_ index:

(5) $${\mathrm{LARG}}_{\mathrm{SC}}={\mathrm{SC}}_{L}+{\mathrm{SC}}_{A}+{\mathrm{SC}}_{R}+{\mathrm{SC}}_{G}$$

The result after the indicated calculations and according to the reflected data is LARG_SC_ = 2.33. This indicates an intermediate implantation value as it is valued on a scale from 1 to 5. It is observed that despite the importance valued in the previous section, now, referring to the implementation level, Lean and Green are the most valued paradigms leaving behind the paradigms Agil and Resilient.

### 3.3. What Level of 4.0 is the LARG to Shipbuilding Supply Chain?

**Figure 5:**
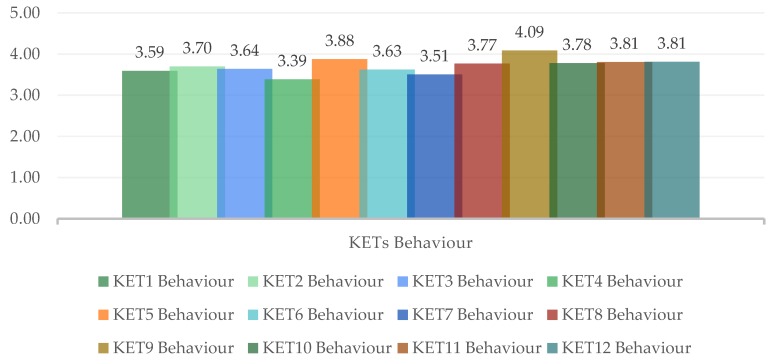
KET’s behavior grouped for each paradigm according to the expert behavior.

In order to know the 4.0 level of the LARG supply chain in shipbuilding, the weighting that the experts answered in this respect is used. Table 4 shows the questions in sections 6 to 17 that highlights the importance of each of the industry 4.0 enabling technologies for the shipbuilding supply chain. In the same way, calculate the mean rating, the weights using Equation (1) and the results shown in Table 5 and Figure 5 are the KETs behavior calculated in the same way as above according to the expert behavior. 

With this data, it is possible to calculate the import of each KET for the SCx (KET-SCx):

(6) $${\mathrm{SC}}_{\mathrm{KETi}}={\mathrm{KETB}}_{i}\cdot w_{\mathrm{KETi}}$$

Being in this case, the index LARG_4.0_ is therefore defined by the following equation:

(7) $${\mathrm{LARG}}_{4.0}=\overset{n}{\underset{i=1}{\sum }}{\mathrm{SC}}_{\mathrm{KETi}}$$

The result in this case of the index value that indicates the level of adaptation to industry 4.0 of the LARG supply chain has turned out to be of LARG_4.0_ = 3.77 indicating that this is a value on a scale of 1 to 5, which is quite interesting as we will analyze it later. Table 6 shows the data needed to calculate the LARG_4.0_ index.

## 4. Discussion

Starting with the study of the importance for the experts of each of the paradigms consulted for the shipbuilding supply chain, the results of the survey indicate that the paradigm with the greatest weight according to the experts is Lean, closely followed by the Agile paradigm, Resilient being the one with a lower weight. Lean is the most widespread and used paradigm for the longest time, which is one of the reasons why it undoubtedly holds the first place in addition to what the paradigm itself offers [101,102]. The Agile paradigm does not seem to be entirely in line with the sector in terms of volume and the great variety of production required in this case as some authors consider [20]. It is true that Resilient is one of the paradigms that most needs SC visibility [23,24]. It is also known that this is a paradigm difficult to get into practice mainly because of the high cost associated and the possibility of bringing the practices related to it close to those of the Lean paradigm. The most impressive of these results is the lower weight of the Green paradigm, as it is considered as a key factor in shipbuilding to increase the competitiveness of the company [103] and improve the energy efficiency [104].

However, studying the implementation each paradigm has, according the supply chain behavior, it is worth mentioning that two of the practices evaluated reached higher mean rate. One of them belongs to the Lean paradigm (PL4) and the other to the Green paradigm (PG2). This shows precisely the declaration on the part of those consulted to prefer for long relationship with suppliers with the benefits that this entail, and second, to be the supporters of the certification of the corresponding regulation over the (PG3) to reduce energy consumption. In conclusion, the predominant paradigms are precisely Lean and Green, and Agile and Resilient being the least. In this case, it coincides totally with previous studies where results are the same for shipbuilding supply chain [40].

If we focus the attention on the value obtained for the LARG index that evaluates how are those paradigms implanted in SC, calculations indicate a value of 2.33. This value, on the same weight scale from 1 to 5, indicates that its implementation is on the way. It also indicates that, according to the consulted experts, the full implementation of the LARG paradigm is needed, mainly because all its benefits has not yet been achieved. This is not the case in other sectors, such as the automotive industry. Comparing the obtained values with the automotive industry (LARG_SC_ = 3.75) [39], it is shown how much the automotive sector is ahead of the shipbuilding sector. The latter demonstrating to have not remained on the sidelines despite the difference between the two sectors. 

Regarding the KET’s, the value obtained is LARG_4.0_ = 3.77. It is higher than that obtained previously (LARG_SC_ = 2.33). Unlike the previous one that indicated the level of implementation of the paradigms to the supply chain, in this case it indicates the level of importance of each KET to each paradigm. Analyzing the results, it is observed that all belong to the same range. KET 9 (Horizontal and Vertical Integration System) exceeds the average value minimally. The reading in this case is that the shipbuilding is committed to adapt to Industry 4.0; however, because of the values obtained, it is not clear which technology can be more interesting to implement sooner.

Focusing on the LARG_4.0_ index, as it is a new creation, it is not possible to compare the index value in itself. However, by targeting the interpretation of that value, it indicates an intermediate level that is in line with the global trend. American companies created the Industrial Internet Consortium (IIC) and some of them opted for the immediate application of the Internet of Things in the shipbuilding sector and it can be said that today shipbuilding has a great demand [105]. In Germany, one of the shipyards that stands out for technological innovation is Meyer-Werft (MW) [106], where the level of use of digital technologies throughout the production process has increased significantly. The identification of parts by radio frequency is another of the fields in which they work, increasing their control and traceability. In Korea, under the so-called "Manufacturing Innovation 3.0 Strategy" innovation is bet on the naval environment of Busan, where the shipyards of Daewoo, Samsung, and Hyundai are working toward the implementation of systems and technologies that are directly aligned with the 4.0 guidelines. Daewoo Shipbuilding and Marine Engineering (DSME) [107] develops systems according to the concept of intelligent factory and integrates technologies that allow optimizing the manufacturing processes; the key for this shipyard is to apply robotics in automating those processes that allow it. The first approaches of Hyundai Heavy Industries (HHI) [108] focused on remote monitoring, evolving into an integrated platform that contemplates fundamental aspects of the ship’s life cycle [109]. The Samsung Heavy Industries (SHI) [110] shipyards quantify in 68% the automation of their productive process, motivated to a great extent by own developments of robotized solutions.

In addition to this first vision, the questionnaire aims to recognize a powerful tool for the leaders that can evaluate which are the practices that best fit in each case.

## 5. Conclusions

With the intention of evaluating where shipbuilding adapts the 4.0 Industry, this article develops an index that allows to know, on the one hand, how its current supply chain is and, on the other hand, how to evaluate the technologies that can allow it to adapt more quickly and efficiently to the 4.0 Industry. The development of both indices has been carried out with the collaboration of experts, both professionals from the shipbuilding sector and academics who have an important connection with this sector.

The importance that experts have given to the different paradigms studied for the supply chain has proved to be the most suitable for the sector in order to achieve a supply chain that contributes greatly to achieve the sustainability of their businesses. The value obtained for the index that indicates the level of implementation is considered satisfactory for an industrial sector in which its pace is different from that of other sectors. However, there is still room for improvement and the calculation of this index could be considered as a tool that can reflect which practices and paradigms could be more interesting to implement.

Finally, the value obtained for the index that indicates the level of adaptation of the supply chain to Industry 4.0, shows us interest as well as some disorientation. In the same way, this can be considered as an opportunity for managers to decide which technologies have priority over others in order to achieve their objectives. This also highlights the need for future research to establish different criteria to improve the index studied.

## Figures and Tables

**Table 1:** Mean rating and weightings of LARG to shipbuilding supply chain.

Paradigms	Questions	Mean Rating	Weight
Lean	How important is Lean paradigm to Shipbuilding Supply Chain	4.22	0.26
Agile	How important is Agile paradigm to Shipbuilding Supply Chain	4.15	0.26
Resilient	How important is Resilient paradigm to Shipbuilding Supply Chain	3.72	0.23
Green	How important is Green paradigm to Shipbuilding Supply Chain	3.93	0.25
	Sum	16.00	1.00

**Table 2:** Questions, mean rating, and weightings of LARG level of implantation to shipbuilding supply chain.

Practices	Reference	Mean Rating	Weight
P_L1_ = Just in time (in the company)	[48]	2.30	0.23
P_L2_ = Just in time (from supplier to company)	[48,49]	2.40	0.24
P_L3_ = Pull Flow	[48]	2.25	0.22
P_L4_ = Supplier relationships/long term business relationship	[50]	3.20	0.32
P_A1_ = Use of IT in design and development activities	[51,52]	2.35	0.26
P_A2_ = Capacity to change delivery times of supplier	[52]	2.35	0.26
P_A3_ = Use of IT in manufacturing activities	[52]	2.15	0.24
P_A4_ = Centralized and collaborative planning	[51]	2.10	0.23
P_R1_ = Procurement strategies that enable the change of suppliers	[53]	1.90	0.28
P_R2_ = Supply chain visibility creation	[24]	1.65	0.22
P_R3_ = Lead time reduction	[23,54]	2.10	0.26
P_R4_ = Development visibility of inventories and demand conditions	[23]	1.75	0.24
P_G1_ = Environmental collaboration with suppliers	[55]	2.15	0.22
P_G2_ = ISO 14001 certification	[28]	3.25	0.33
P_G3_ = To reduce energy consumption	[56]	2.10	0.21
P_G4_ = To reduce or recycling materials and packaging	[57]	2.40	0.24
	Sum	36.40	

**Table 3:** Data LARG_SC_ index.

Paradigm	1	2	3	4	5	6	7	8	9	10	11	12	13	14	15	16	17	18	19	20	PB_x_	w_x_	SC_x_
Lean	3.55	2.63	3.00	4.55	3.00	2.73	1.78	2.13	2.32	3.08	2.32	1.54	2.23	2.32	2.63	2.78	3.09	2.87	1.54	1.86	2.60	0.26	0.68
Agile	2.00	2.79	2.99	1.77	2.00	1.53	1.76	2.53	3.23	1.74	2.00	2.26	2.74	1.50	3.00	2.03	2.00	2.53	2.47	2.00	2.24	0.26	0.58
Resilient	2.26	1.76	2.54	1.28	2.00	1.00	2.00	1.72	1.80	2.28	1.78	2.28	2.28	2.00	2.78	2.00	2.00	1.00	1.28	1.26	1.87	0.23	0.43
Green	2.57	3.35	4.33	2.90	3.11	1.57	1.87	2.57	2.12	2.03	1.45	2.11	2.76	2.76	3.64	2.66	2.33	2.90	2.09	2.11	2.56	0.25	0.63

**Table 4:** Key enabling technologies and supply chain paradigms questionnaire.

Key Enabling Technologies	Lean (L)	Agile (A)	Resilient (R)	Green (G)
K1: Additive Manufacturing	Large scale production in small batches, with a focus on the customer and the creation of more with less waste [59]	Customized products and processes [18]	Manufacturing products close to the customers geographic location reduces response time [60]	Technologies allowing techniques for the reuse and recycling of urban waste [61]
K2: Big Data	Improved information flows that enable the supply chain to operate with reduced inventory and rapid customer response [23]	Provide a single view of market trends, customer purchasing patterns and maintenance cycles, ways to reduce the costs and enable more targeted business decisions [62]	Assist in making decisions regarding pricing, optimization, reduction of operational risk, and improvement of the delivery of products and services [62]	Minimize the risk inherent in hazardous materials, associated carbon emissions, and economic cost [63]
K3: Cloud Computing	Supporting information management during the life cycle of the digital product [62]	Facilitating sharing of resources and participants collaboration throughout the supply chain lifecycle [64]	Prevent potential vulnerabilities in the cooperation of supply chain actors [65]	Evaluate the ecological performance of the supplier under economic and environmental criteria [66]
K4: Augmented Reality	Provide advance shipping instructions and times [67]	Shorten the learning curve [68]	Possibility of tracking the product [69]	Promote disintermediation [70]
K5: Autonomous Robots	Improve work cycle efficiency [71]	Increase production flexibility [72]	Improve control of interrupt detection [73]	Minimize transport time [74]
K6: Automated Vehicles	Helps achieve leaner processes [75]	Improve supply chain performance by planning and controlling movements [76]	Enable online algorithm adaptation with real-time response [77]	Guarantee the performance of environmental efficiency [78]
K7: Blockchain	Increase the efficiency, reliability and transparency of the entire supply chain, optimize input processes (ability to ensure the immutability of data) and public accessibility of data flows [79]	Provide strong process controls aligned to the interests of all operators involved [80]	Enables transactions with a unified, transparent record keeping system [81]	Allows information easily disseminated to multiple parties involved, compiling and verification of information, control of environmental quality of materials, time management for new product development projects and coordination of participants [82]
K8: Cybersecurity	Improve efficiency within the business structure [83]	Analyze the requirements of the regulations of the different countries involved [84]	Allows to take steps to build resilience [85]	Optimize overall resource, energy consumption, provider operating expenses minimizing potential loss of security in the event of a successful attack on any virtual machine [86]
K9: Horiz. & Vert. Integ. System	Improve flow and vertical integration of all departments resulting in reduced delivery time of the product or service [70]	Facilitate ability to deliver products and services on time [87]	Strengthen synergy of their networks, execute activities across the entire value chain in an intelligent manner [88]	Allow the establishment of connections with the systems of information [88]
K10: Artificial intelligence	To pursue cost reduction through the use of tools such as Just in Time [89]	Provide real-time, adaptive visibility and traceability [90]	Identifies the most resilient suppliers [91]	Select the best suppliers according to economic and environmental criteria [92]
K11: Internet of Things	Improve the efficiency and quality of production and distribution [93]	Make prompt decisions and accelerate material flows by integrating and exchanging information flows to improve effectiveness and efficiency [94]	Formulate strategies to mitigate risks [95]	Improve decision making efficiency for inventory management [96]
K12: Simulation	Facilitate a design that allows to consider pull systems [97]	Measure the efficiency of the process [98]	Increase your complexity and vulnerability to disruptions [99]	Evaluate alternative policies for long-term capacity planning [100]

**Table 5:** Mean rating and weight of each KET studied.

Key Enabling Technologies	Lean		Agile		Resilient		Green	
	Mean Rating	Weight	Mean Rating	Weight	Mean Rating	Weight	Mean Rating	Weight
K1	3.79	0.25	4.21	0.28	3.21	0.21	3.79	0.25
K2	4.05	0.26	3.79	0.24	3.84	0.25	3.89	0.25
K3	3.95	0.26	3.79	0.25	3.89	0.25	3.74	0.24
K4	3.58	0.25	3.47	0.24	3.79	0.26	3.47	0.24
K5	4.26	0.26	4.21	0.26	4.11	0.25	3.84	0.23
K6	3.79	0.25	4.21	0.27	3.68	0.24	3.63	0.24
K7	3.68	0.25	3.63	0.25	3.84	0.26	3.63	0.25
K8	4.16	0.26	3.84	0.24	4.16	0.26	3.79	0.24
K9	4.47	0.26	4.47	0.26	4.32	0.25	4.05	0.23
K10	4.16	0.26	4.00	0.25	3.89	0.24	3.89	0.24
K11	4.11	0.26	4.11	0.26	3.89	0.24	3.95	0.25
K12	4.05	0.25	4.26	0.26	3.95	0.25	3.84	0.24

**Table 6:** LARG_4.0_ index data.

KET’s	1	2	3	4	5	6	7	8	9	10	11	12	13	14	15	16	17	18	19	KETB_x_	w_KETi_	SC_KETi_
KET1	4.00	3.81	3.07	4.28	3.56	3.51	4.07	3.79	2.47	4.25	3.00	4.07	3.53	3.79	3.75	4.49	3.82	4.57	4.07	3.78	0.08	0.31
KET2	3.00	4.32	4.09	3.22	3.24	5.00	4.23	3.22	3.45	3.92	4.00	3.91	5.00	4.00	4.14	3.68	4.08	3.63	3.86	3.89	0.08	0.32
KET3	3.00	4.08	4.32	3.92	3.22	5.00	4.00	4.00	3.13	3.68	3.24	4.00	5.00	4.00	3.68	4.15	4.76	2.54	3.08	3.83	0.08	0.31
KET4	3.45	3.77	3.68	2.22	3.76	3.85	3.68	3.37	3.14	4.46	3.68	3.77	4.00	4.00	3.00	4.31	4.31	2.77	2.54	3.57	0.08	0.27
KET5	4.00	4.24	4.00	4.00	3.78	4.37	3.82	4.15	3.91	4.77	3.46	4.00	4.00	4.00	4.00	4.54	4.08	4.46	4.00	4.08	0.09	0.36
KET6	3.55	4.32	3.54	3.93	3.00	4.78	4.68	3.93	3.23	3.68	3.46	4.00	4.46	4.00	3.68	3.47	4.01	3.46	3.33	3.82	0.08	0.31
KET7	3.77	3.67	3.32	2.23	3.00	5.00	3.22	3.69	3.69	3.91	3.68	3.77	4.23	4.00	3.68	4.76	4.31	3.09	3.08	3.69	0.08	0.29
KET8	5.00	4.45	3.77	3.31	3.76	5.00	3.67	4.21	3.45	4.32	3.68	3.92	4.00	4.00	3.22	4.46	3.23	4.00	3.92	3.97	0.08	0.34
KET9	4.00	4.32	3.91	4.68	4.00	5.00	4.68	4.37	3.54	4.46	4.15	4.77	5.00	4.00	3.24	4.46	5.00	4.78	3.45	4.31	0.09	0.39
KET10	4.00	4.56	3.00	4.76	3.46	4.15	3.22	3.32	3.54	4.76	4.37	3.32	5.00	4.00	4.00	4.68	3.46	4.00	4.00	3.98	0.08	0.34
KET11	4.00	4.22	3.00	3.09	4.00	4.78	5.00	3.68	3.55	4.00	4.37	3.46	5.00	3.32	4.00	4.54	3.46	4.23	4.46	4.01	0.09	0.34
KET12	3.00	3.77	3.23	5.00	3.22	4.78	4.24	4.15	3.33	4.32	4.37	3.24	4.24	4.00	4.00	4.14	4.23	5.00	4.00	4.01	0.09	0.34
